# Rarity to Remission: Complete Response of Primary Breast Diffuse Large B‐Cell Lymphoma With Chemoimmunotherapy

**DOI:** 10.1002/ccr3.71891

**Published:** 2026-01-20

**Authors:** Abhisek Jha, Vivek Ghosh, Shristi Gupta, Samana Oli, Asmita Rayamajhi, Dhiraj Gupta, Anish Luitel, Barun Khanal, Aabish Dahal, Sarada Khadka

**Affiliations:** ^1^ Department of Clinical Oncology Birat Medical College and Teaching Hospital (BMCTH) Morang Nepal; ^2^ Department of Breast & Endocrine Surgery BP Koirala Institute of Health Sciences (BPKIHS) Dharan Nepal

**Keywords:** chemoimmunotherapy, diffuse large B‐cell lymphoma (DLBCL), non‐Hodgkin's lymphoma, primary breast lymphoma, remission

## Abstract

Primary Non‐Hodgkin's lymphoma of the breast is rare, accounting for < 0.5% of breast cancers and ~2% of extranodal lymphomas. It often presents as a painless lump, mimicking carcinoma and complicating diagnosis. Case PresentationWe report a 65‐year‐old post‐menopausal, hypertensive, and diabetic woman with a gradually enlarging left breast mass. Imaging revealed a suspicious lesion. Core biopsy suggested poorly differentiated carcinoma, but immunohistochemistry confirmed diffuse large B‐cell lymphoma (DLBCL), activated B‐cell subtype, with high Ki‐67 (~90%). PET/CT showed localized disease. She received six cycles of CHOP chemotherapy, with rituximab added from the second cycle. Treatment was well tolerated. Follow‐up PET/CT and biopsy demonstrated complete metabolic and pathological remission. ConclusionPrimary breast DLBCL is rare and easily misdiagnosed as carcinoma. Immunohistochemistry is therefore crucial for correct diagnosis. R‐CHOP chemoimmunotherapy is the cornerstone of treatment.

## Key Clinical Message

Primary breast lymphoma (PBL) is an uncommon extranodal lymphoma which often mimics carcinoma, leading to diagnostic delays. Immunohistochemistry is essential for accurate diagnosis. The optimal treatment modality is still under investigation because of the rarity of this disease. Chemoimmunotherapy with rituximab, cyclophosphamide, doxorubicin, vincristine, and prednisone (R‐CHOP) can achieve complete remission, highlighting its effectiveness in managing localized PBLs.

## Graphical abstract

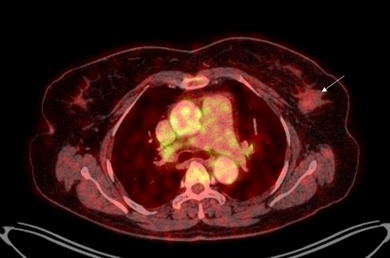

## 1 Introduction

Primary Non‐Hodgkin's lymphoma of the breast is a rare disease that was first reported by Bobrontina et al., in 1959 [1]. It includes 0.1%–0.5% of all malignant breast cancers and approximately 1.7%–2.2% of extranodal Non‐Hodgkin's lymphomas. They affect all age groups with a propensity for bilateral breast involvement in the younger population and unilateral breast involvement in the older ones. Primary breast lymphomas (PBL) usually present with solitary or multiple painless palpable breast lumps. Nipple discharge, nipple or skin retraction are rare presentations. Classical B symptoms of Hodgkin's lymphoma are rarely seen in Primary Breast Lymphomas. The most common histological subtype of Non‐Hodgkin's lymphoma is diffuse large B‐cell lymphoma (DLBCL) followed by follicular and mucosal‐associated lymphoid tissue lymphomas [2]. The diagnosis is usually based on biopsy and immunohistochemistry. Treatment modalities previously involved surgery with recent protocols focusing more on chemoradiation therapy with great success [2, 3]. Here, we present a very rare case of primary DLBCL, activated B‐cell subtype of the left breast in a hypertensive and diabetic woman treated successfully with R‐CHOP regimen.

## 2 Case History/Examination

A 65‐year‐old post‐menopausal woman, a known case of hypertension and Type II diabetes mellitus on regular treatment, presented to the oncology clinic with a painless, gradually progressive left breast lump which she first noticed 2 months prior to presentation. She denied any history of breast pain, nipple discharge, nipple retraction, overlying skin changes, or prior breast trauma. She denied any history of significant unintentional weight loss, night sweat, or fever. However, she reported that her father had died of gastric carcinoma. There was no personal or family history of breast or ovarian malignancy. On local breast examination, there was presence of a single, well‐defined, painless, palpable lump of approximately 1 × 2 cm in the upper outer quadrant of the left breast. The lump was hard, immobile, and associated with tethering of surrounding tissue. The overlying skin appeared normal, with no erythema, peau d'orange, ulceration, or nipple involvement. Contralateral breast examination was unremarkable. Bilateral axillary, supraclavicular, and cervical lymph nodes were not palpable. General physical and systemic examination was unremarkable.

## 3 Methods (Differential Diagnosis, Investigations, and Treatment)

**FIGURE 1:**
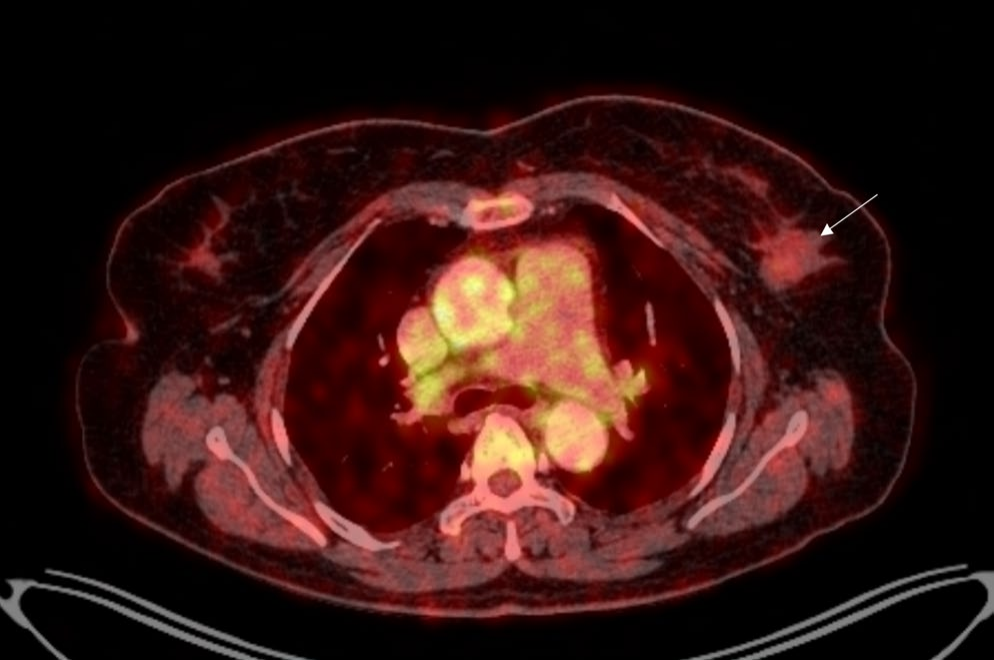
Baseline positron emission tomography/computed tomography (PET/CT) scan showing faintly FDG avid heterogenously enhancing soft tissue density lesion in the left breast (white arrow).

Mammographic evaluation identified a suspicious lesion within the breast, classified as BI‐RADS category 4, indicating a moderate likelihood of malignancy and warranting histopathological assessment. A core needle biopsy of the lesion demonstrated diffuse sheets of neoplastic cells with a dense lymphoid background, initially interpreted as a poorly differentiated (Grade III) invasive carcinoma of no special type (NST). Contrast Enhanced Computed Tomography of Neck, Chest and Abdomen was done for metastatic workup. It showed a heterogeneously enhancing soft tissue density lesion with spiculated margin measuring 2.4 × 1.2 × 1.9 cm along with tiny foci of calcification within the upper outer quadrant of the left breast consistent with the malignant breast mass. The scan also revealed homogeneously enhancing left level I axillary lymph nodes, largest measuring ~8 × 6 mm along with prevascular, paratracheal and para‐aortic lymph nodes. To further characterize the lesion, immunohistochemical analysis was performed. The tumor cells exhibited strong expression of CD20, BCL6, and MUM1, with additional positivity for CMYC and weak BCL2. Markers including TdT, CD10, and CD138 were negative. The proliferation index (Ki‐67) was approximately 90%, indicative of a highly proliferative neoplasm (Table 1). The immunophenotypic profile supported the diagnosis of high‐grade B‐cell Non‐Hodgkin's lymphoma (NHL), most consistent with diffuse large B‐cell lymphoma (DLBCL), activated B‐cell subtype. These findings revised the initial impression of invasive carcinoma and underscored the hematolymphoid nature of the neoplasm. Whole body Positron Emission Tomography/Computed Tomography (PET/CT) was done to assess metastasis of the primary tumor. It revealed faintly F‐18‐Fluoro‐2‐deoxy‐D‐glucose (FDG) avid heterogenously enhancing soft tissue density lesion in the left breast with a SUV max of 1.1 (Figure 1) with faintly FDG avid subcentimetric sized left axillary lymph nodes, largest measuring 10 × 7 mm with no scan evidence of any significant active disease in rest of the visualized segment of the body. After staging the disease as I_E_, the patient was planned for six cycles of CHOP regimen with addition of rituximab starting from the second cycle due to economic constraints. Each cycle was administered at a 3‐week interval. The dosages were as per standard recommendations (rituximab @375 mg/m^2^, cyclophosphamide@750 mg/m^2^, doxorubicin@50 mg/m^2^, vincristine@1.4 mg/m^2^ on day 1) intravenously along with oral prednisone (@45 mg/m^2^ from day 1 to day 5) in each cycle. The adjunct treatment such as ondansetron, ranitidine, hydrocortisone and olanzapine were also administered to minimize minor side effects of chemoimmunotherapy.

**TABLE 1:** Immunohistochemical profile of the left breast lesion suggestive of primary breast lymphoma—Diffuse large B‐cell lymphoma (PB‐DLBCL).

Marker	Expression	Interpretation
CD20	Strong positive	B‐cell lineage
BCL6	Strong positive	Germinal center/activated B‐cell marker
MUM1	Strong positive	Non‐germinal center phenotype
CMYC	Positive	Proliferation‐related marker
BCL2	Weak positive	Anti‐apoptotic marker
TdT	Negative	Immature lymphoid cells absent
CD10	Negative	Germinal center marker absent
CD138	Negative	Plasma cell marker absent
Ki‐67	~90%	Highly proliferative neoplasm

## 4 Results and Conclusions (Outcome and Follow‐Up)

**FIGURE 2:**
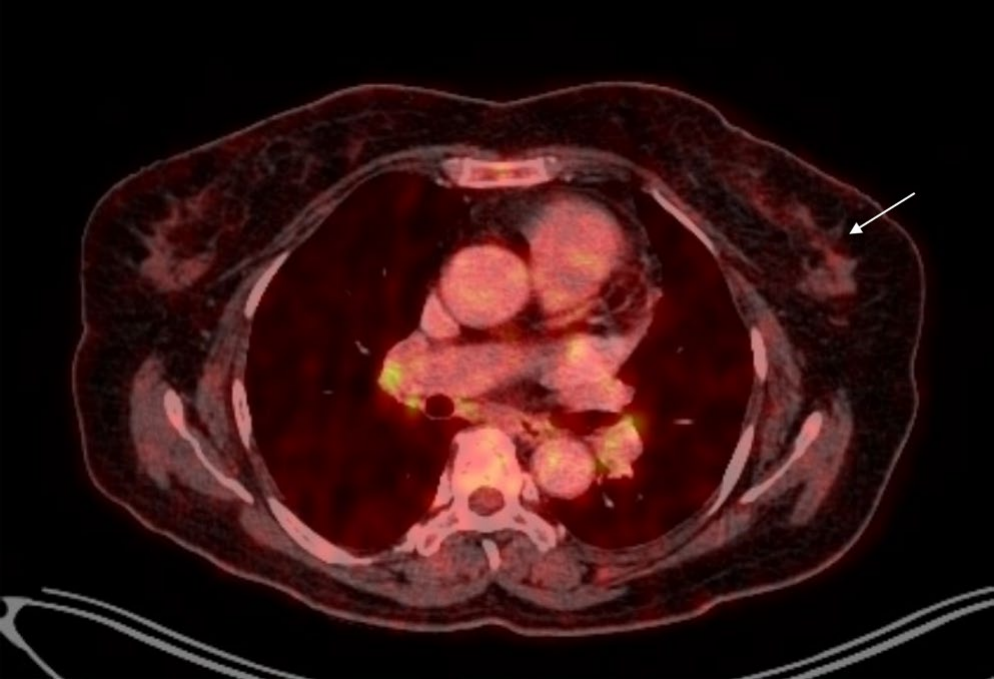
Response assessment positron emission tomography/computed tomography (PET/CT) post 6 months of treatment showing non‐FDG avid heterogeneously enhancing soft tissue density lesion in the left breast with resolution of metabolic activity (white arrow).

The patient tolerated chemoimmunotherapy without any major hematological and systemic complications. At 6 months post‐treatment, follow‐up ^18^F‐FDG PET‐CT scan of the whole body demonstrated a non‐FDG avid heterogeneously enhancing soft tissue density lesion in the left breast (Figure 2) with non‐FDG avid subcentimetric sized left axillary lymph nodes, largest measuring 9 × 5 mm with no active disease elsewhere. In comparison to previous PET‐CT, there was resolution of metabolic activity (no abnormal FDG uptake) of the left breast lesion and left axillary nodes with minimal reduction in size of the few left axillary nodes confirming a complete metabolic response. Also, a repeat core biopsy of the left breast revealed fibro‐collagenous hyalinized stroma—consistent with treatment effect without residual malignancy revealing a pathological complete response. The patient is kept under regular follow up with needful investigations and clinical examination periodically.

## 5 Discussion

Among hematological cancers, lymphomas are diagnosed frequently, but primary breast lymphoma is a very rare diagnosis comprising 0.1%–0.5% of all malignant breast cancers and about 1.7%–2.2% of extranodal lymphomas. It usually affects women in their fifth and sixth decade of life [1]. The rarity of the primary breast lymphoma is usually attributed to the fact that breast contains very little lymphoid tissue [4].

Around 90% cases of PBL are B‐cell derived, most commonly DLBCL followed by follicular, marginal zone, and Burkitt variants [5]. The predominant T‐cell variant is Anaplastic Large cell Lymphoma, commonly associated with textured breast implants. Primary breast diffuse large B‐cell lymphoma (PB‐DLBCL), one of the common but aggressive subtypes, usually presents with gradually enlarging, painless, palpable breast lump/s, breast enlargement, and axillary lymphadenopathy thereby mimicking breast carcinoma and complicating the diagnosis. It can sometimes manifest with systemic B symptoms such as fever, weight loss, and fatigue. The proposed risk factors for PB‐DLBCL include post‐menopausal status, chronic inflammatory and autoimmune disease, pregnancy, and lactation [5]. In accordance with the literature, in our case, the post‐menopausal woman presented with gradually enlarging, painless, solitary breast lump for 2 months. However, our patient did not report any of the systemic B symptoms.

Primary breast lymphoma (PBL) is diagnosed and staged using Wiseman and Liao's criteria: the lymphoma must present primarily in the breast, show close association with breast tissue, lack evidence of disseminated disease within 6 months of diagnosis and adequate quality of histopathological specimen. Concurrent axillary lymph node involvement is considered part of the PBL spectrum [6]. All of these criteria were fulfilled in our case, warranting a diagnosis of PBL—stage I_E_.

Imaging findings in PBL are usually non‐specific, often mimicking other breast malignancies. Mammography typically shows smooth oval mass usually with well‐circumscribed margins. Calcifications, architectural distortion, nipple retraction, or spiculations are usually not seen in association with this malignancy. Ultrasonography generally demonstrates reduced internal echogenicity with posterior acoustic enhancement—reflecting high cellularity, whereas dynamic magnetic resonance imaging shows fast early phase enhancement—suggesting hypervascularity [7]. Histology is essential for the diagnosis of PB‐DLBCL, in which it reveals diffuse infiltrate of large neoplastic B cells with round to oval or lobulated nuclei and frequent prominent nucleoli. Similarly, in immunohistochemistry, tumor cells express B‐cell markers (CD20, CD79α, PAX5), and most often exhibits a non‐germinal center phenotype (CD10−, MUM1+) along with high proliferation index (Ki‐67 > 80%) [5]. In our case, the specimen revealed diffuse sheets of neoplastic cells in dense lymphoid background—interpreted as poorly differentiated invasive carcinoma. However, on immunohistochemical analysis, the tumor cells exhibited strong expression of CD20, BCL6, and MUM1 along with high proliferation index (Ki‐67‐90%) thereby emphasizing the role of immunohistochemistry in diagnosing, characterizing PB‐DLBCL, differentiating it from invasive carcinomas and directing the course of therapy. ^18^F‐FDG PET‐CT is crucial for the initial diagnosis of PB‐DLBCL where it shows high FDG avid lesions as well as for accurately assessing systemic involvement, therapeutic response and recurrence on follow‐up [8, 9]. In a similar vein, the left breast lesion as well as the left level I axillary nodes in our case, demonstrated faint FDG avidity with SUV max of 1.1 before chemoimmunotherapy whereas the follow‐up scan at 6 months post‐treatment showed non‐avid lesion in the left breast and non‐ avid reduced left axillary nodes with no disease activity elsewhere in the body—reflecting the importance of systemic PET scans in disease evaluation, staging and monitoring of therapy and recurrence. Additionally, lower baseline PET metabolic activity, including SUV max, may predict improved R‐CHOP response in DLBCL and could explain the exceptional outcome in our activated B‐cell case [10].

Management of PB‐DLBCL usually involves more than three cycles (six cycles being standard) of anthracycline‐based chemotherapy regimens, particularly CHOP, with the addition of rituximab—shown to improve central nervous system (CNS) relapse rates [11].

Surgery is generally restricted to diagnostic purposes such as core needle or excisional biopsy as extensive surgery carries a high rate of morbidity without any proven advantage [11]. Radiotherapy serves as an important consolidation approach, particularly in node‐negative (stage I_E_) disease, whereas combination therapy is recommended in cases of high‐grade tumor, axillary node, and CNS involvement. Routine CNS prophylaxis is often unnecessary with optimal combined‐modality therapy but should be considered in high‐risk patients, such as those with poor performance status or elevated LDH levels. The prognosis of PB‐DLBCL depends upon staging, use of radiotherapy or combination therapy, tumor size as well as nodal status and is often poor (5‐year survival rate of 40%–80%) due to frequent CNS relapses [11, 12, 13]. In a parallel manner, our patient underwent six cycles of CHOP regimen, with the addition of rituximab from the second cycle. Although the FLYER trial demonstrated non‐inferiority of four cycles of CHOP with six doses of rituximab compared to six cycles of R‐CHOP in young patients with aggressive B‐cell lymphoma, its findings are not directly applicable to older patients (> 60 years) or those with extranodal disease. Given the patient's age, primary breast involvement, and activated B‐cell subtype, a full course of six cycles of R‐CHOP was preferred [14]. Given the anatomical proximity of the heart to the left breast and the documented risk of radiation‐induced cardiac toxicity associated with left‐sided thoracic irradiation, and considering the patient's age and comorbidity profile, six cycles of R‐CHOP were chosen instead of abbreviated chemotherapy followed by radiotherapy alone, to minimize long‐term cardiopulmonary risk [15, 16]. Although the activated B‐cell (non‐GCB) subtype of DLBCL is typically associated with inferior outcomes, CHOP—especially with the addition of rituximab—can achieve complete remission in localized, low‐tumor burden presentations, such as in our patient [17, 18]. The patient on repeat core biopsy and systemic PET scan at 6 months follow‐up showed no residual malignancy and metabolically active disease in the body—demonstrating complete response. As the follow‐up period remains relatively short, the patient continues to be monitored with regular clinical assessments and imaging, underscoring the importance of extended follow‐up in PBL due to potential for late relapse.

## 6 Conclusion

Primary breast diffuse large B‐cell lymphoma (PB‐DLBCL) with activated B‐cell phenotype is a rare and aggressive malignancy that can mimic breast carcinoma, making accurate diagnosis challenging. Immunohistochemistry is essential for differentiating it from other breast neoplasms and guiding therapy. Combined chemoimmunotherapy with R‐CHOP, supplemented by radiotherapy when indicated, can achieve complete remission in localized disease, even in biologically adverse subtypes. This case underscores the importance of early recognition, precise immunohistochemical evaluation, and appropriate systemic therapy in achieving favorable outcomes in PB‐DLBCL. The case also aims to highlight gaps in current literature regarding follow‐up protocols in cases of primary breast lymphomas.

## 7 Limitation

Economic constraints to add rituximab from the first cycle itself have been the major limitation in this case.

## Author Contributions

**Abhisek Jha:** writing – original draft, writing – review and editing. **Vivek Ghosh:** conceptualization, supervision, writing – review and editing. **Shristi Gupta:** writing – original draft. **Samana Oli:** writing – review and editing. **Asmita Rayamajhi:** supervision. **Dhiraj Gupta:** supervision. **Anish Luitel:** writing – original draft. **Barun Khanal:** writing – original draft. **Aabish Dahal:** writing – original draft. **Sarada Khadka:** supervision.

## Funding

The authors have nothing to report.

## Consent

Written informed consent was obtained from the patient to publish this report in accordance with the journal's patient consent policy.

## Conflicts of Interest

The authors declare no conflicts of interest.

## Data Availability

The authors have nothing to report.
